# Teetering on a liver’s edge: a case report highlighting clinical decision-making in thrombocytopenia

**DOI:** 10.1186/s12885-019-6302-0

**Published:** 2019-11-06

**Authors:** Adam Yan, Laura Erdman, Lillian Sung, Stacey Bernstein

**Affiliations:** 1 Department of Paediatrics, The Hospital for Sick Children, 555 University Avenue, Toronto, ON M5G 1X8 Canada; 2 Department of Hematology and Oncology, The Hospital for Sick Children, Toronto, Canada

**Keywords:** Immune thrombocytopenic purpura, Acute lymphoblastic leukemia, Thrombocytopenia, Bone marrow analysis

## Abstract

**Background:**

This report illustrates the importance of a detailed history and physical exam and careful analysis of hematologic parameters when diagnosing ITP. This case demonstrates that even with subtle deviations from typical ITP findings one must promptly reevaluate the diagnosis. This case also highlights the importance of peripheral smear review by an expert in pediatric hematopathology.

**Case presentation:**

A previously healthy 10 year-old Asian boy presented with 2 months of easy bruising. Review of systems was negative for any constitutional symptoms. On examination, he appeared well but had numerous large ecchymoses. He had no appreciable lymphadenopathy or splenomegaly. The liver was palpable 1.5 cm below the costal margin. A complete blood count (CBC) showed: platelets = 17 × 109/L, hemoglobin = 128 g/L, white blood cell count = 5.43 × 109/L, and neutrophils = 1.63 × 109/L. A blood smear was reported as normal. Urate was 370 umol/L and lactate dehydrogenase (LDH) was 803 U/L. The child was admitted with a presumptive diagnosis of immune thrombocytopenic purpura (ITP) and treated with intravenous immunoglobulin. The following day, the blood smear was reviewed by a hematopathologist who identified blasts. A bone marrow aspiration (BMA) confirmed the diagnosis of precursor B-cell acute lymphoblastic leukemia.

**Conclusion:**

In children presenting with suspected ITP, leukemia should always be considered. A BMA was historically performed on all patients with presumed ITP to rule out leukemia. In 2011, the American Society of Hematology (ASH) stopped recommending routine BMA in patients suspected of having ITP. ASH advises in cases with unusual findings on history, physical examination or CBC, it is reasonable to perform a BMA. Our patient had mild hepatomegaly, which may have qualified him for a BMA. He also had an elevated LDH and urate, which are not listed as criteria for BMA by ASH but were considered atypical for ITP by the clinical team. A literature search did not reveal any primary data assessing these markers.

While corticosteroids are a first line treatment in ITP, they must be reserved for when clinicians are confident that the patient does not have leukemia. Steroid administration prior to diagnosing leukemia results in delayed diagnosis and may increase the risk of complications and decrease survival.

## Background

Thrombocytopenia is one of the most common hematologic abnormalities encountered in pediatrics [1]. Thrombocytopenia is defined as a platelet count less than 150 × 10^9^ /L irrespective of age [1]. The clinical manifestations of thrombocytopenia include petechiae, purpura, gingival bleeding, epistaxis, menorrhagia, gastrointestinal bleeding, hematuria and central nervous system hemorrhage. The mechanisms of thrombocytopenia include diminished platelet production, shortened platelet life span, platelet sequestration and platelet loss or dilution (Table 1) [1].

**Table 1:** Differential diagnosis of thrombocytopenia

A) Diminished Platelet Production	-Marrow infiltration
	-Marrow injury
	-Infection
	-Genetic syndromes (rare)
B) Shortened Platelet Life Span -Immune Mediated -Nonimmune Mediated	-Immune thrombocytopenic purpura (ITP)
	-Neonatal alloimmune thrombocytopenia
	-Infection
	-Heparin induced thrombocytopenia
	-Drug or vaccine induced
	-Systemic autoimmune disease
	-Disseminated intravascular coagulation
	-Hemolytic uremic syndrome
	-Thrombotic thrombocytopenic purpura
	-Major surgery or trauma
	-Infection
C) Platelet Sequestration or Pooling	-Hypersplenism
	-Kasabach-Merritt phenomenon
	-Chronic liver or storage disease
	-Portal vein thrombosis
	-Von Willebrand disease (type 2B and platelet-type)
D) Platelet dilution	-Massive transfusion of packed red blood cells or whole blood

## Case presentation

A previously healthy, 10-year old male, Asian child presented to the emergency department with a two-month history of easy bruising. No episodes of mucocutaneous bleeding or petechiae were reported, other than one brief episode of self-limited epistaxis 2 weeks prior to presentation. There was no antecedent history of a viral illness, immunizations, drug exposure, recent travel or known sick contacts.

Review of systems was negative for any constitutional symptoms including bone pain, fevers, night sweats, anorexia, or weight loss. There was no family history of bleeding disorders, childhood malignancy, or autoimmune conditions.

On initial examination the child was afebrile, and appeared well. He had good energy and color. He had numerous large ecchymoses over the bony prominences of his shoulders and elbows, and he had several ecchymoses on his shins. He had no appreciable lymphadenopathy or splenomegaly. The liver was palpable approximately 1.5 cm below the right costal margin. The remainder of his physical exam was within normal limits.

Bloodwork was done in the emergency department. An initial complete blood count showed: platelets = 17 × 10^9^/L, hemoglobin = 128 g/L, white blood cell count = 5.43 × 10^9^/L, lymphocytes = 2.29 × 10^9^/L, and neutrophils = 1.63 × 10^9^/L. A blood smear was reported as normal pending final hematopathologist review. Urate was 370 umol/L (normal = 100–277) and lactate dehydrogenase (LDH) was 803 U/L (normal = 432–700). Calcium, phosphate, potassium, creatinine, PTT and INR were all within normal limits. A chest radiograph showed no mediastinal mass or hilar adenopathy.

The child was admitted to hospital with a presumptive diagnosis of immune thrombocytopenic purpura (ITP). A hematology consult was requested. Therapeutic options were discussed. Given the concern for possible leukemia due to the palpable liver, elevated urate and elevated LDH, the recommended course of action was intravenous immunoglobulin (IVIG) therapy and avoidance of corticosteroids. Post IVIG therapy, the platelets rose from 17 to 58 × 10^9^ / L.

The following day, the peripheral blood smear was reviewed by a hematopathologist who identified the presence of blasts. Flow cytometry of the peripheral blood showed 12% blasts which were positive for CD10, CD19 and CD20, and a bone marrow aspiration confirmed the diagnosis of precursor B-cell acute lymphoblastic leukemia (ALL). The child was transferred to the oncology service for further evaluation and treatment, including a spinal tap. He was classified as having high risk central nervous system 2 ALL based on age and presence of blast cells in his CSF. Chemotherapy was initiated as per the Children’s Oncology Group protocol AALL1131.

## Discussion and conclusion

ITP can be classified based on the duration of thrombocytopenia. If the thrombocytopenia resolves within 12 months of onset it is classified as acute ITP and if it persists beyond 12 months it is classified as chronic ITP. Acute ITP is the most common cause of pediatric thrombocytopenia, affecting 5/100,000 children annually [1]. ITP is an autoimmune disorder characterized by immunologic destruction of platelets resulting in isolated thrombocytopenia in an otherwise well looking child without lymphadenopathy or hepatosplenomegaly [2]. The median age of presentation with ITP is 69 months [3]. The incidence of ITP peaks between 2 and 5 years of age with a second smaller peak in adolescents [4]. Primary ITP is defined as a platelet count less than 100 × 10^9^ / L in the absence of other causes or disorders that may cause thrombocytopenia [5]. Alternatively ITP may occur secondary to other autoimmune disorders (e.g., systemic lupus erythematosus, anti-phospholipid syndrome, Evans syndrome), viral infections (e.g., cytomegalovirus, hepatitis c, varicella zoster, human immunodeficiency virus) [5] or drugs (e.g., carbamazepine, quinine, and vancomycin) [2, 6].

In children presenting with suspected ITP, leukemia should be considered in the differential diagnosis. Although leukemia is classically associated with hematopoietic abnormalities beyond isolated thrombocytopenia, a bone marrow evaluation was historically performed on all patients with a presumed diagnosed of ITP to rule out leukemia prior to starting therapy. However, a number of retrospective studies called this practice into question [7, 8]. A review of 2239 children enrolled in two Pediatric Oncology Group acute lymphoblastic leukemia clinical trials showed that only 1 child presented with isolated thrombocytopenia, who had no blast cells on the blood smear, hemoglobin > 110 g/L and an absolute neutrophil count > 1.5 × 10^9^ / L. On physical examination, that child had marked hepatosplenomegaly, and thus was unlikely to be misclassified as typical ITP [7]. Subsequently, a review of confirmatory bone marrow analyses performed in 322 children presenting with typical features of acute ITP found zero cases of leukemia [8].

In 2011, the American Society of Hematology (ASH) revised its position statement on ITP, no longer recommending routine bone marrow evaluation in patients suspected of having typical ITP [5]. Both ASH and the American Association of Pediatrics (AAP) advise that in the case of unusual findings on history or physical examination such as fevers, weight loss, fatigue, bone pain, lymphadenopathy or hepatosplenomegaly, or unusual hematologic findings such as abnormal white or red blood cell counts, it is reasonable to perform a bone marrow examination [5, 9]. While ITP is more in children between 2 and 5 years of age and in adolescents, there is no recommendation to alter your diagnostic work-up for children presenting with what appears to be ITP at an atypical age [4].

Our patient had mild hepatomegaly, which may have qualified him for a bone marrow examination even if the peripheral smear was negative. He also had an elevated LDH and urate, which are not listed as criteria for bone marrow evaluation by the ASH or AAP guidelines but were considered as atypical for ITP by the clinical team caring for this patient. Some experts have recommended bone marrow examination if the LDH and urate are elevated in pediatric ITP [9]. However, a literature search did not reveal any primary data assessing these serum markers A recent study in adult patients, showed that urate is commonly elevated in patients with ITP [10]. Further work is needed to understand the significance of a high LDH and urate in pediatric ITP.

The initial management of ITP can include both observation and pharmacologic therapies. Between 80 and 90% of ITP is self-limited, with patients making a full and permanent recovery within 12 months without any treatment [11]. Children over the age of 10 have an increased risk of progressing to chronic ITP with 47.3% having a platelet count < 150 × 10^9^/L 6 months after diagnosis [4]. Patients with platelet counts below 10–20 × 10^9^/L are at increased risk for significant bleeding and this often prompts clinicians to offer pharmacologic interventions to raise the platelet count expeditiously. Initial pharmacologic options include IVIG or a short course of oral corticosteroids [5]. Costs, risk of bleeding and parental preferences factor into decision making [12].

While corticosteroids are one of the first line pharmacologic agents in ITP, they must be reserved for cases where clinicians are confident that the patient does not have leukemia. Steroid administration prior to the diagnosis of leukemia results in delayed diagnosis and may increase the risk of complications and decrease event-free survival [13].

In our case, the patient presented to a quaternary care pediatric hospital with excellent access to laboratory technology and specialists. Recognition of atypical features consisting of mild hepatomegaly, and unexplained elevation of LDH and urate prompted clinicians to recommend IVIG and not corticosteroids as the initial treatment.

This report illustrates the importance of a detailed history and physical exam and careful analysis of presenting hematologic parameters when diagnosing ITP. Even subtle deviations from findings in typical ITP should prompt clinicians to reevaluate the diagnosis. This case also highlights the importance of peripheral smear review by an expert in pediatric hematology/oncology to ensure that blast cells are not missed. In this case, careful review ensured that the patient was fully investigated and did not receive steroids. It is important to emphasize that bone marrow analysis is not required for cases of typical ITP.

## Data Availability

We do not have any raw data supporting our findings.

## Abbreviations

AAP: American Association of Pediatrics
ALL: Acute lymphoblastic leukemia
ASH: American Society of Hematology
BMA: Bone marrow aspirate
CBC: Complete blood count
ITP: Immune thrombocytopenic purpura
IVIG: Intravenous immunoglobulin
LDH: Lactate dehydrogenase (LDH)
